# Preliminary evaluation of the FastCAP for users of the Nurotron cochlear implant

**DOI:** 10.3389/fnins.2024.1523212

**Published:** 2025-01-07

**Authors:** Xue-Ying Yang, Sui Huang, Qian-Jie Fu, John Galvin, Bing Chen, Ji-Sheng Liu, Duo-Duo Tao

**Affiliations:** ^1^The First Affiliated Hospital of Soochow University, Suzhou, China; ^2^Zhejiang Key Laboratory of Neuroelectronic and Brain Computer Interface Technology, Hangzhou, China; ^3^David Geffen School of Medicine, University of California, Los Angeles, Los Angeles, CA, United States; ^4^House Institute Foundation, Los Angeles, CA, United States; ^5^Eye and ENT Hospital, Fudan University, Shanghai, China

**Keywords:** cochlear implant, electrically evoked compound action potential, threshold level, comfortable loudness level, Nurotron

## Abstract

**Background:**

Electrically evoked compound action potential (ECAP) can be used to measure the auditory nerve’s response to electrical stimulation in cochlear implant (CI) users. In the Nurotron CI system, extracting the ECAP waveform from the stimulus artifact is time-consuming.

**Method:**

We developed a new paradigm (“FastCAP”) for use with Nurotron CI devices. In electrically evoked compound action potential in fast mode (FastCAP), N recordings are averaged directly on the CI hardware before data transmission, significantly reducing data transmission time. FastCAPs and ECAPs were measured across five electrodes and four stimulation levels per electrode. The FastCAP stimulation rate (33.3 Hz) is also faster than the ECAP rate (2.5 Hz).

**Results:**

Results showed strong correlations between ECAPs and FastCAPs for N1 latency (*r* = 0.84, *p* < 0.001) and N1 amplitude (*r* = 0.97, *p* < 0.001). Test-retest reliability for FastCAPs was also high, with intraclass correlation coefficients of *r* = 0.87 for N1 latency (*p* < 0.001) and *r* = 0.96 for N1 amplitude (*p* < 0.001). The mean test time was 46.9 ± 1.4 s for the FastCAP and 340.3 ± 6.3 s for the ECAP. The FastCAP measurement time was significantly shorter than the ECAP measurement time (*W* = −210.0, *p* < 0.001). FastCAP thresholds were significantly correlated with behavioral thresholds in 7/20 participants and with comfortable loudness levels in 11/20 participants. The time required to measure FastCAPs was significantly lower than that for ECAPs. The FastCAP paradigm maintained the accuracy and reliability the ECAP measurements while offering a significant reduction in time requirements.

**Conclusion:**

This preliminary evaluation suggests that the FastCAP could be an effective clinical tool to optimize CI processor settings (e.g., threshold stimulation levels) in users of the Nurotron CI device.

## 1 Introduction

Neural response measurement (NRM) involves detecting and quantifying the electrically evoked compound action potential (ECAP) originating from the auditory nerve within the cochlear implant (CI). ECAPs can be used to objectively measure aspects of electric stimulation such as current spread and loudness growth. ECAPs can be used to guide clinical fitting, especially in children who cannot provide subjective feedback. ECAPs have been used to infer neural health at stimulation sites across the cochlea (e.g., Schvartz-Leyzac and Pfingst, 2016; Yuan et al., 2022).

The ECAP typically consists of a negative peak (N1) within a time window of 0.2–0.4 ms after stimulus onset, followed by a positive peak (P2) occurring around 0.6–0.8 ms after stimulus onset (Brown et al., 1990; Abbas et al., 1999). The ECAP provides valuable information about the auditory nerve’s response to electrical stimulation (Cullington, 2000). As an electrophysiological response that can be directly measured through the CI system, the ECAP is an objective tool that can be used to assess the physiological state and function of the auditory nerve, as well as the effectiveness of stimulation across CI electrodes. This holds significant clinical importance for CI users and increases understanding of the nervous system’s physiological responses to electrical stimulation (Briaire and Frijns, 2005).

One important aspect of the ECAP research is optimization of the ECAP measurement itself. ECAP measurements require optimization to address stimulus artifact, individual anatomical variations, etc. Enhancing ECAP techniques will improve the accuracy of auditory nerve response measurements, benefiting both clinical management of CI users and advancing understanding of neural responses to electrical stimulation. ECAP optimization is essential to improve the reliability and precision of response data processing and analysis. Brown et al. (1990) developed the Forward Masking Auditory Nerve Response Telemetry (FM-ANRT) to minimize stimulus artifacts when measuring neural responses in CI users. ECAP recordings are obtained for four distinct stimulation conditions:

(A) Probe alone. A single biphasic pulse is used as the probe. The recorded data includes stimulus artifact, neural response to the probe, and system noise.
(B) Masker + probe. A masker and a probe pulse are presented in sequence with an inter-pulse interval. If the inter-pulse interval is sufficiently brief to fall within the refractory period of the auditory nerve, the neural response to the probe is predominantly influenced by the preceding masker. In the majority of cases, an interval of less than a specific range, for example, 0.3–0.5 ms, is deemed to be sufficiently short (Morsnowski et al., 2006). With a sufficiently short inter-pulse interval, the neural response to the probe is dominated by the preceding masker. The recorded data encompasses masker artifact, masker neural response, stimulus artifact, and system noise.
(C) Masker alone. The recording encompasses masker artifact, masker neural response, and system noise.
(D) No stimulation. The recording captures only the system noise.

**FIGURE 1:**
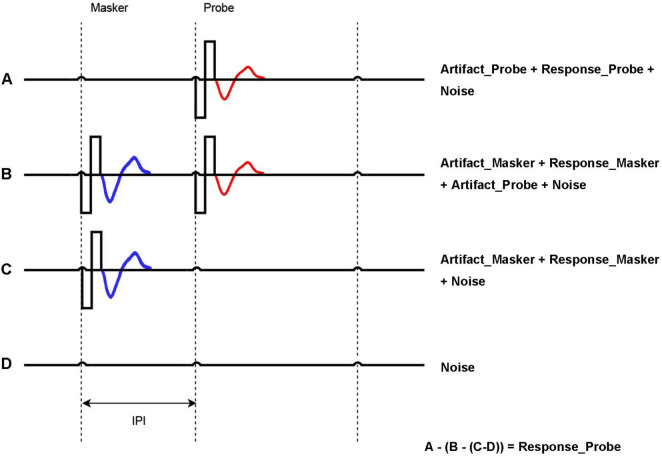
Forward masking approach to acquire ECAP. A = Probe artifact + Probe response (red) + Noise; B = Masker artifact + Masker Response (blue) + Probe response (red) + Probe artifact + Noise; C = Masker artifact + Masker Response (blue) + Noise; D = Noise. Probe response = A − (B − C) − D. The inter-pulse interval (IPI) is the time between Masker and Probe, which is key to generating the masking effect.

Figure 1 shows the stimulation and recording process for A, B, C, and D steps, respectively. Once recordings are obtained for these four steps, the stimulus response is obtained by calculating: A – (B – (C– D)). This allows for accurate measurement of the probe’s response and sensitivity of the auditory nerve (Abbas et al., 1999).

While FM-ANRT effectively addresses the issue of stimulus artifact, it also has its drawbacks. One major drawback is the method requires somewhat long measurement times due to the four steps. For traditional ECAP measures, the sequence of four steps (A, B, C, and D) is executed consecutively, with recording data being sent from the implant to the fitting software in the computer after completing each step (e.g., Nurotron device) or each sequence (e.g., Cochlear device). Due to the large volume of data transmission between the computer and implant, this mode tends to be slow. In the present study, we developed and evaluated a new algorithm (“FastCAP”) by averaging the neural recording on the CI hardware to minimize the data transmission that limits the speed of the traditional ECAP measurement.

**FIGURE 2:**
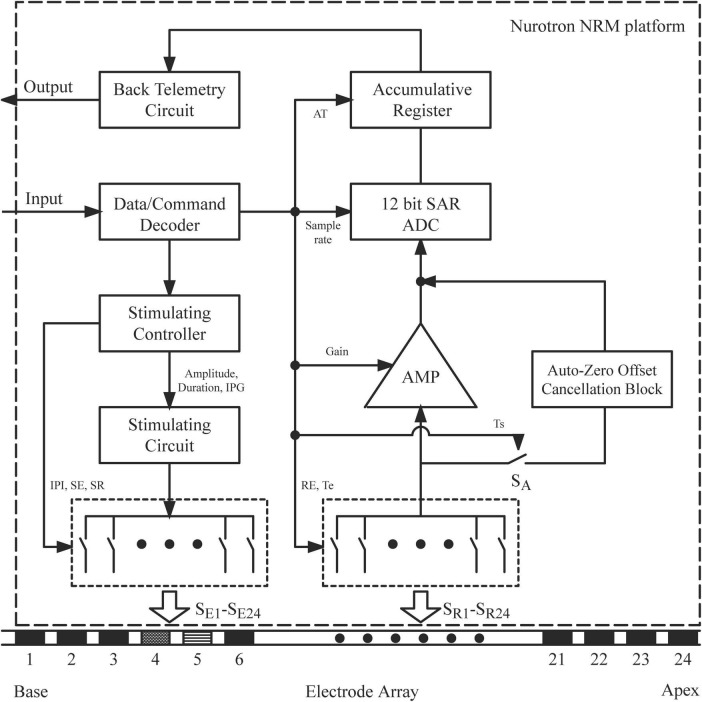
Block diagram of the Nurotron NRM configurable platform. IPG, inter-phase gap; IPI, inter-pulse interval; SR, stimulation rate; SE, stimulating electrode; SR, recording electrode; AT, accumulative time; SAR, successive approximation register; ADC, analog-to-digital converter; AMP, amplifier; Te, access delay; Re, recording electrode; Ts, system delay; Sa, switch for Auto-Zero offset Cancellation Block.

The electrically evoked compound action potential in fast mode (FastCAP) algorithm was developed using the NRM configurable platform developed by Nurotron Biotechnology Inc. to be specifically used with the CS-20A implant. The platform allows for manipulation of the parameters of the NRM system to conduct electrophysiology research. Figure 2 shows the block diagram of the Nurotron NRM configurable platform. The output data from the analog-to-digital converter is stored in the accumulative register. Once the system measurement reaches the set accumulative time, all data are transmitted by the back telemetry circuit. Details such as the specific hardware configuration and other intricate technical specifications have been moved to Supplementary Appendix 1.

We also investigated associations between FastCAPs and behavioral measures of threshold levels (T-levels) and comfortable loudness levels (C-levels) (Brown et al., 1996, 1998, 2000; Abbas et al., 1999; Hughes et al., 2000). This is an important clinical consideration as NRM could be used instead of behavioral measures of T- and C-levels for clinical fitting of speech processors. The ECAP threshold – the minimum electrical stimulation current amplitude required to elicit measurable ECAP responses – is commonly used to predict T-levels (Zarowski et al., 2020; Franck and Norton, 2021; Holstad et al., 2009; Allam and Eldegwi, 2019). Moderate to strong correlations between ECAP thresholds and T- or C-levels have been observed across studies, with the correlation coefficient r ranging from 0.5 to 0.9 (Di Nardo et al., 2003; Smoorenburg et al., 2002; Cullington, 2000; Polak et al., 2005; Allam and Eldegwi, 2019; Chao et al., 2023).

In summary, the FastCAP methodology represents a significant advancement in CI management, offering a less time-consuming and more efficient approach to NRMs. By drastically reducing the time required for ECAP assessments, FastCAP addresses a critical bottleneck in clinical workflows, making it particularly advantageous in busy clinical settings. This efficiency not only enhances the practicality of CI programming but also allows clinicians to allocate more time to patient care and personalized adjustments. As a result, FastCAP stands out as a transformative tool for optimizing auditory rehabilitation and advancing the precision and accessibility of CI technology.

## 2 Materials and methods

### 2.1 Participants

Twenty native Chinese (7 females and 13 males) users of the Nurotron CS-20A device participated in the Experiment 1. These participants were recruited from the First Affiliated Hospital of Soochow University in Suzhou, from 1 July 2023 to 15 September 2023. The mean age across all participants was 24.45 ± 16.95 years (range: 1.0–56.0 years). The mean duration of deafness was 4.45 ± 4.91 years (range: 1.0–21.0 years), and the mean CI experience was 0.43 ± 0.28 years (range: 0.1–1.0 years). Participant demographic information is shown in Supplementary Table 1.

Another 20 native Chinese users of the Nurotron CS-20A CI device (10 males and 10 females) participated in the Experiment 2; none of these CI users participated in Experiment 1. These participants were recruited from the First Affiliated Hospital of Soochow University in Suzhou, from 1 October 2023 to 25 December 2023. The mean age at testing was 29.25 ± 17.51 years (range: 5.0–62.0 years), the mean duration of deafness was 7.88 ± 5.58 years (range: 1.0–24.0 years), and the mean CI experience was 0.32 ± 0.14 years (range: 0.1–0.6 years). Participant demographic information is shown in Supplementary Table 2.

All participants underwent cone-beam computed tomography after the CI surgery to confirm proper implantation. Electrode impedances were measured before testing to ensure functionality.

### 2.2 Ethics statements

The present study was reviewed and approved by Institutional Review Board in The First Affiliated Hospital of Soochow University, Suzhou, China (approval number: 2023244). The date of ethical approval is 19 June 2023. Informed consent for study participation and data publication was obtained from all participants in this study. For adult participants, written consent was collected using signed forms prior to their participation. For participants under 18 years of age, consent was obtained from their parents or legal guardians, and minors provided assent where appropriate.

### 2.3 Experiment 1: FastCAP vs. ECAP

#### 2.3.1 FastCAP protocol development

**FIGURE 3:**
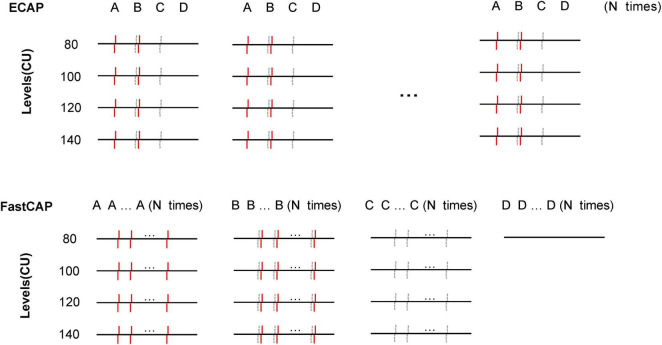
Schematic illustration of the traditional ECAP **(top)** and FastCAP method **(bottom)**. In the ECAP, *N* recordings of A, B, C, and D are transmitted to the PC. In the FastCAP, *N* recordings are averaged for A, B, C, and D before being transmitted to the PC; D (noise) is only recorded once as it is not expected to change over successive iterations. The red solid lines and gray dashed lines indicate the masker and probe, respectively.

In the traditional forward masking subtraction method used to extract the ECAP, is each step is performed *N* times at a 2.5 Hz rate (Figure 3). To speed up the ECAP measurement, an accumulative register is used in FastCAP to store the measurement data in the Nurotron CS-20A CI device. Successive recordings are averaged and saved in the register (maximum of 128 measurements) before data transmission. In FastCAP, step A is run *N* times, followed by *N* iterations of steps B and C (Figure 3); to further reduce measurement time, step D is only measured once for each test electrode, as this will not change over subsequent recordings. The essential difference between the traditional ECAP and the FastCAP is that in the ECAP, data of each recording are transmitted for steps A, B, C, and D *N* times, and then averaged in the computer, while in the FastCAP, data for steps A, B, C, and D are averaged separately across *N* recordings on the CI hardware before transmission. Overall, the traditional ECAP requires a total of 4 × *N* data transmissions while the FastCAP only needs 4 data transmissions for *N* iterations. This greatly reduces transmission time between the implant and clinical interface.

#### 2.3.2 FastCAP and ECAP parameters and measurements

Electrically evoked compound action potential in fast mode and ECAP measurements were performed at four stimulation levels (80, 100, 120, and 140 clinical units, or CUs) for five test electrodes equally spaced across the array (1, 7, 13, 19, and 24). The recording electrodes were located adjacent to the test electrodes (2, 8, 14, 20, and 23). For FastCAP, the interphase gap was 10 μs, the masker-probe inter-pulse interval was 350 μs, the pulse phase duration was 50 μs, the amplifier gain was 44 dB, the stimulation rate was 33.3 Hz, and the number of iterations (*N*) was 10. For ECAP, the parameters were the same, except that the stimulation rate was 2.5 Hz; the lower rate was necessary to accommodate the slow data transmission after each sequence of steps. The pulses used in this study are cathodic-leading symmetrical biphasic pulses, ensuring consistent stimulation and recording across all electrodes. Both the stimulation and recording are relative to an extracochlear monopolar ground, providing a stable reference for measurements. For the forward-masking paradigm, the masker pulse is delivered at a higher CUs level than the probe pulse (10 CUs). This offset ensures effective masking, where the neural response to the probe is dominated by the preceding masker (Hughes et al., 2001). The masker pre-activates nerve fibers, driving them into a refractory state to suppress responses to the probe stimulus. Setting the masker intensity 10 CUs higher than the probe could ensures full neural activation and optimal masking, preventing incomplete suppression that could reduce ECAP measurement accuracy. Hughes et al. (2001) found that a 10 CU difference achieves saturation, where further increases in masker intensity do not improve masking, making this the ideal condition for accurate ECAP measurements. CUs are a standardized measure used to quantify stimulation levels in CIs, based on the amplitude of the stimulation current and pulse phase duration. The system access delay for beginning recording at 25 μs. For each participant, FastCAPs and ECAPs were each measured within a single session (Test 1); FastCAPs were re-measured again on the following day for test-retest reliability (Test 2).

Electrically evoked compound action potential in fast modes and ECAPs were calculated according to the subtraction methods described above (section “1 Introduction”; Figure 2). Latency was defined as the time between the start of the recording and the appearance of the first negative peak (N1). This was determined via visual observation, which involved direct observation of the response waveforms.

### 2.4 Experiment 2: FastCAP thresholds vs. behavioral T- and C-levels

#### 2.4.1 Behavioral measurements of T-levels and C-levels

Testing was conducted in an electromagnetically shielded chamber where the background noise was <35 dBA. The NuroSound software was used to provide auditory stimulation, and T- and C-levels were obtained for electrodes 1, 7, 13, 19, and 24. Stimuli used for behavioral measurement in the clinical fitting were 500 ms, charge-balanced, anodic-first, symmetric, biphasic pulse trains. The stimulation rate was 680 pulses per second, the pulse phase duration was 50 μs, the inter-phase gap was 10 μs and the stimulation mode was monopolar (MP1 + 2). For measurement of T-levels, the stimulus level was first linearly increased from 0 CUs until the participant indicated that they heard a sound. Next, stimulation level was increased to be audibly above threshold, then decreased until the participant indicated that they could no longer hear the sound. Next, the level was further decreased to a definite sub-threshold level, then increased until the participant indicated that they heard a sound; this current level was deemed the T-level. C-levels were measured by increasing the stimulation level until the participant indicated that sound was comfortably loud. The step size for adjusting T- or C-levels was 1 CU.

#### 2.4.2 FastCAP threshold measurements

The FastCAP parameters were the same as in Experiment 1. Behavioral thresholds were used to set the initial FastCAP stimulation levels. FastCAP thresholds were measured by adjusting the stimulation level in 5 CU steps. For a given stimulation level, if a FastCAP response was observed, the stimulation was reduced until a response could no longer be observed, and then increased until the FastCAP response could be observed again. Visual inspection was used to determine FastCAP responses. The threshold was defined as the lowest CU where a response could still be visualized. The approximate size of the noise floor was 2 μV.

### 2.5 Statistical methods

The response data were subjected to non-parametric tests and Pearson correlation analysis using SPSS (ver. 25.0; Armonk, NY); *p* values < 0.05 were considered to be significant; where appropriate, *p* values were adjusted to correct for multiple comparisons.

### 2.6 Data availability

The dataset supporting the findings of this study is openly available in Mendeley Data, https://data.mendeley.com/datasets/cxhy5rd3zy/1.

## 3 Results

### 3.1 Experiment 1

#### 3.1.1 FastCAP vs. ECAP

**FIGURE 4:**
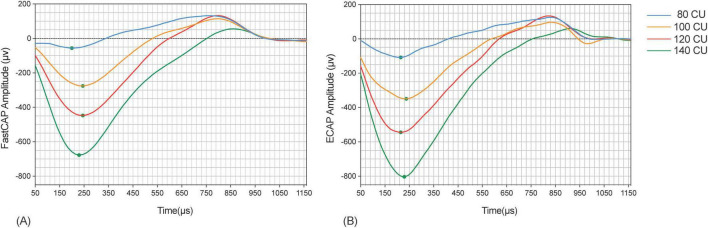
Example FastCAP **(A)** and ECAP **(B)** waveforms for participant 64 on electrode 19 for the four stimulus levels. The ECAP waveforms are color-coded as follows: blue for a stimulus level of 80 CU, orange for 100 CU, red for 120 CU, and green for 140 CU.

Figure 4 shows example FastCAP and ECAP waveforms for E19 from participant S64. In this example, the mean N1 latency across all stimulus levels was 228 ± 32 μs for the FastCAP and 233 ± 33 μs for ECAP. The N1 amplitude increased from 130–145 to 172–274 μV across stimulus levels for the FastCAP, and from 179–192 to 188–373 μV across stimulus levels for the ECAP. The different stimulation rates (2.5 Hz for ECAP vs. 33 Hz for FastCAP) may have contributed to the differences in amplitudes observed with the different stimulation levels.

**FIGURE 5:**
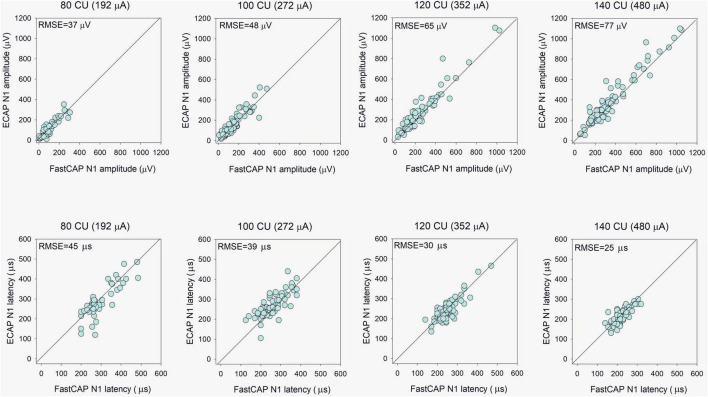
**(Top)** Evoked compound action potential N1 amplitude as a function of FastCAP N1 amplitude (*n* = 20). **(Bottom)** ECAP N1 latency as a function of FastCAP N1 latency. Plots are shown for 80, 100, 120, and 140 CU stimulus presentation levels; data for all participants and all electrodes are shown in each panel. The diagonal lines show unity; values above the line indicate higher values for the ECAP and values below the line indicate higher values for the FastCAP. The mean RMSE between the ECAP and FastCAP values are shown in the top left of each panel.

The top panels of Figure 5 show ECAP N1 amplitude as a function of FastCAP N1 amplitude at the four stimulation levels; the raw data can be found in Supplementary Data Sheet 1. Mean and standard deviation within and across stimulation levels are shown in Table 1. Note that FastCAPs and/or ECAPs could not be obtained for all participants, electrodes, and or levels; data are shown only where both FastCAP and ECAPs could be obtained. Across all participants, stimulation levels, and electrodes, a Wilcoxon signed rank test showed that, N1 amplitude was significantly higher with ECAP than with FastCAP (*W* = 19,313, *p* < 0.001); however, there was no significant difference in N1 latency between ECAP and FastCAP (*W* = −3051, *p* = 0.187). Strong correlations were observed between FastCAP and ECAP N1 amplitudes and latencies within and across the four stimulation levels (Table 1). The root mean square error (RMSE) between the ECAP and FastCAP N1 amplitudes steadily increased with stimulation level. The RMSE between ECAP and FastCAP N1 latencies steadily decreased with stimulation level.

**TABLE 1:** Mean and standard deviation of N1 amplitude and latency values within and across the four clinical unit (CU) stimulation levels with the FastCAP and ECAP methods.

			80 CU	100 CU	120 CU	140 CU	All
Amplitude (μV)	FastCAP	Mean	108	153	239	377	230
		SD	76	100	183	270	207
	ECAP	Mean	120	169	265	413	255
		SD	82	109	200	287	224
	Correlation	*r*	0.90	0.90	0.96	0.97	0.97
		*p*	<0.001	<0.001	<0.001	<0.001	<0.001
	RMSE		37	48	65	77	61
Latency (μs)	FastCAP	Mean	295	259	248	222	252
		SD	72	57	50	36	58
	ECAP	Mean	285	268	242	216	250
		SD	82	56	52	34	62
	Correlation	*r*	0.85	0.77	0.84	0.78	0.84
		*p*	<0.001	<0.001	<0.001	<0.001	<0.001
	RMSE		45	39	30	25	34

Data were pooled across participants and stimulated electrodes. The Pearson correlation coefficients between the FastCAP and ECAP data are shown within and across stimulation levels. The root mean square error (RMSE) between the FastCAP and ECAP values is shown within and across stimulation levels.

#### 3.1.2 Time consumption: FastCAP vs. ECAP

The mean test time (across participants and electrodes) was 46.9 ± 1.4 s for the FastCAP and 340.3 ± 6.3 s for the ECAP. Across all participants, stimulation levels, and electrodes, a Wilcoxon signed rank test showed that the FastCAP measurement time was significantly shorter than the ECAP measurement time (*W* = −210.0, *p* < 0.001). The time difference was primarily due to the faster stimulation rate used in FastCAP (33.3 Hz) than in ECAP (maximum = 2.5 Hz).

#### 3.1.3 Test-retest reliability for FastCAP

**FIGURE 6:**
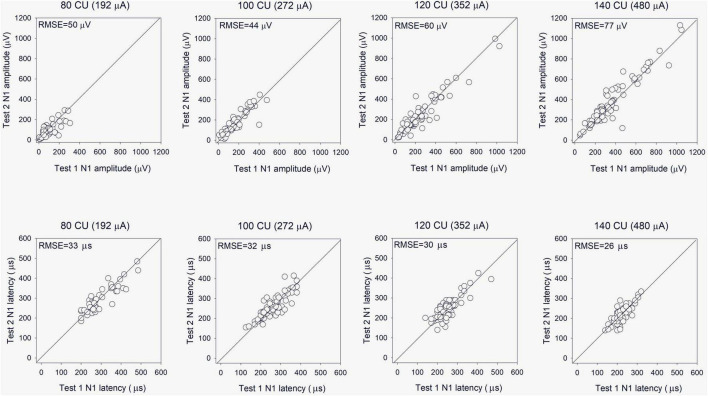
**(Top)** Test 2 FastCAP N1 amplitude as a function of Test 1 FastCAP N1 amplitude (*n* = 20). **(Bottom)** Test 2 FastCAP N1 latency as a function of Test 1 FastCAP N1 latency. Plots are shown for 80, 100, 120, and 140 CU stimulus presentation levels; data for all participants and all electrodes are shown in each panel. The diagonal lines show unity; values above the line indicate higher values for Test 2 and values below the line indicate higher values for Test 1. The mean RMSE between the Test 2 and Test 1 values are shown in the top left of each panel.

Electrically evoked compound action potential in fast modes were obtained on two consecutive days to test reliability. The top panels of Figure 6 show Test 2 N1 amplitude as a function of Test 1 N1 amplitude at the four stimulation levels. Mean and standard deviation within and across stimulation levels are shown in Table 2; the raw data can be found in Supplementary Data Sheet 1. Note that FastCAPs for both Test 1 and Test 2 could not be obtained for all participants, electrodes, and or levels; data are shown only where both FastCAPs for both Test 1 and Test 2 could be obtained. Across all participants, stimulation levels, and electrodes, a Wilcoxon signed rank test showed no significant difference between FastCap Test 1 and Test 2 in terms of N1 amplitude (*W* = 2,414, *p* = 0.379) or N1 latency (*W* = 2,589, *p* = 0.219).

**TABLE 2:** Mean and standard deviation of FastCAP N1 amplitude and latency values within and across the four clinical unit (CU) stimulation levels for Test 1 and Test 2.

			80 CU	100 CU	120 CU	140 CU	All
Amplitude (μV)	Test 1	Mean	107	153	236	381	231
		SD	75	99	182	266	207
	Test 2	Mean	102	151	230	380	226
		SD	66	100	175	259	201
	Correlation	*r*	0.76	0.90	0.94	0.96	0.96
		*p*	<0.001	<0.001	<0.001	<0.001	<0.001
	RMSE		50	44	60	77	60
Latency (μs)	Test 1	Mean	296	260	250	222	252
		SD	75	57	51	36	58
	Test 2	Mean	298	259	246	216	251
		SD	83	56	51	41	63
	Correlation	*r*	0.87	0.84	0.84	0.79	0.87
		*p*	<0.001	<0.001	<0.001	<0.001	<0.001
	RMSE		33	32	30	26	30

Data were pooled across participants and stimulated electrodes. The Pearson correlation coefficients between the FastCAP and ECAP data are shown within and across stimulation levels. The RMSE between Test 1 and Test 2 values is shown within and across stimulation levels.

### 3.2 Experiment 2: FastCAP thresholds vs. behavioral T- and C-levels

**FIGURE 7:**
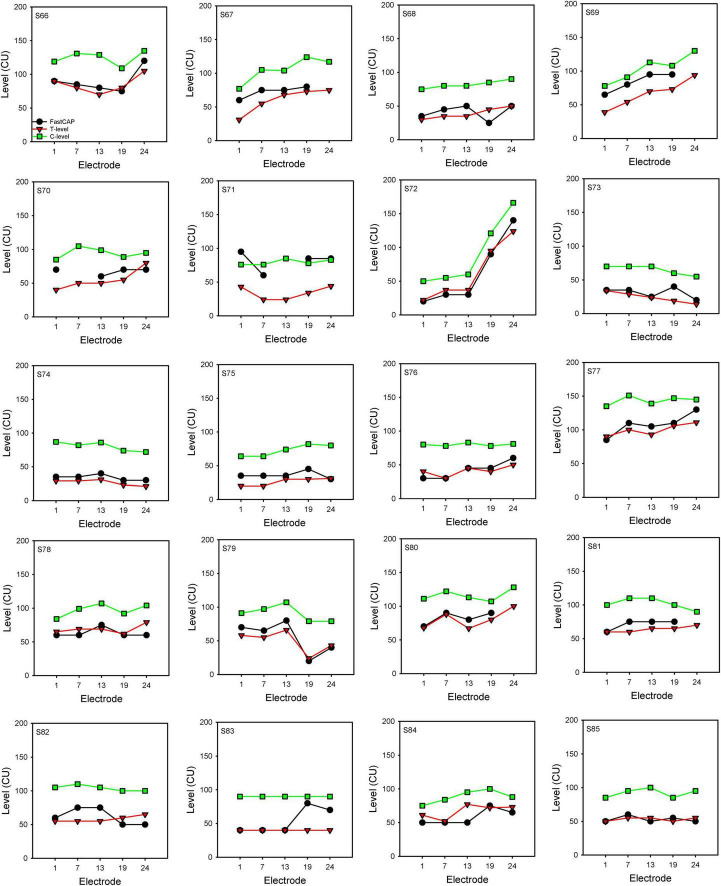
FastCAP thresholds, T- and C-levels for individual participants. FastCAP thresholds (black), T-levels (red), and C-levels (green) for individual participants for each of the test electrodes.

**FIGURE 8:**
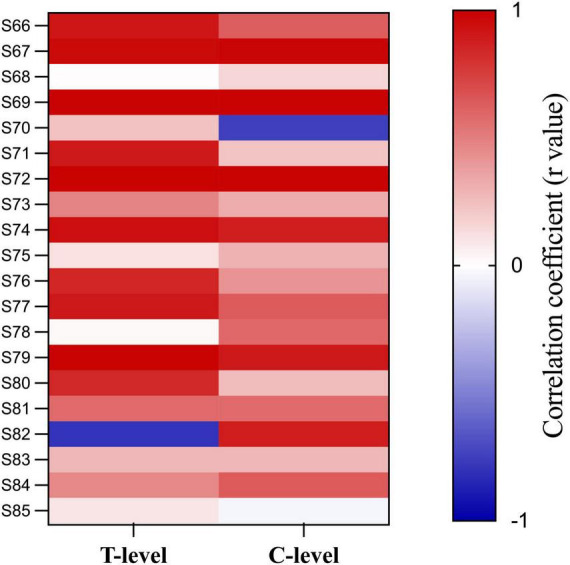
Heat map of the correlation between FastCAP thresholds, T- and C-levels. Each cell represents the correlation coefficient for individual participants.

Figure 7 shows FastCAP thresholds, T-levels, and C-levels (all in CU) for individual participants for each of the test electrodes. Note that FastCAP thresholds could not be obtained in all participants at all electrodes; the raw data can be found in Supplementary Data Sheet 1. In general, FastCAP thresholds were elevated relative to T-levels. Within each participant, the mean difference between FastCAP thresholds and T-levels was calculated across electrodes. Across all participants, FastCAP thresholds were on average 7.9 ± 13.7 CUs higher than T-levels. Across all data, a Wilcoxon signed rank tests showed that FastCAP thresholds were significantly higher than T-levels (*W* = −2,141.0, *p* < 0.001) and significantly lower than C-levels (*W* = 4,430.0, *p* < 0.001). In some cases, FastCAP thresholds could not be obtained for some electrodes in some participants. These instances were due to the FastCAP threshold not being reachable with a tolerable loudness level on E24 for S67, E24 for S69, E7 for S70, and E13 for S71. The E24 electrode impedance for S77 and S78 was so high that the FastCAP threshold could not be obtained. Additionally, we included a heatmap illustrating the correlation between the FastCAP threshold and T/C levels (Figure 8).

In some participants, the patterns of the FastCAP thresholds, behavioral T- and C-levels across electrodes (threshold “profile”) were quite similar. For each participant, the fidelity of the threshold profile between the FastCAP and behavioral T-levels was quantified using different approaches (Table 3). The mean RMSE across electrodes was calculated between the FastCAP and T-levels without or with adjustment for DC bias (the mean shift between the FastCAP and T-level profiles). RMSE without adjustment represents the error when including the absolute differences between the FastCAP and T-level profiles. RMSE with adjustment represents the error between profiles after normalizing to the mean thresholds across electrodes. The normalized root mean square error (NRMSE) was calculated according to the following equation (Otto, 2019):


(1) NRMSE=

$$\frac{\sqrt{\sum_{i=1}^{N}{\left(\left(x_{i}-\sum_{j=1}^{N}x_{j}/N\right)-\left(y_{i}-\sum_{j=1}^{N}y_{j}/N\right)\right)}^{2}/N}}{\left(\sqrt{\sum_{i=1}^{N}{\left(x_{i}-\sum_{j=1}^{N}x_{j}/N\right)}^{2}/N}+\sqrt{\sum_{i=1}^{N}{\left(y_{i}-\sum_{j=1}^{N}y_{j}/N\right)}^{2}/N}\right)/2}$$

**TABLE 3:** Different approaches to quantify the similarity between the FastCAP and behavioral profiles across electrodes.

Participant	RMSE between FastCAP and T-level (without DC adjustment)	RMSE between FastCAP and T-level (with DC adjustment)	NRMSE between FastCAP and T-level	Correlation coefficient between FastCAP and T-level	Correlation coefficient between FastCAP and C-level
S66	8.66	7.07	0.46	0.91*	0.63
S67	18.30	9.31	0.68	0.96*	0.98*
S68	12.25	12.08	1.27	0.01	0.16
S69	24.80	1.64	0.11	0.99*	0.99*
S70	18.20	14.31	1.30	0.24	−0.75
S71	45.50	6.75	0.56	0.90	0.23
S72	8.75	8.69	0.18	0.99*	0.99*
S73	10.15	7.35	0.91	0.48	0.32
S74	7.52	1.36	0.32	0.94*	0.88*
S75	11.84	6.65	1.19	0.11	0.30
S76	6.71	6.63	0.66	0.85	0.42
S77	11.37	8.07	0.65	0.90*	0.64
S78	10.07	8.23	1.25	0.02	0.59
S79	9.64	7.70	0.38	0.99*	0.90*
S80	8.32	4.87	0.50	0.84	0.26
S81	10.31	5.45	1.05	0.58	0.58
S82	15.17	14.63	1.72	−0.80	0.88*
S83	22.36	17.44	1.79	0.28	0.28
S84	13.62	10.22	0.94	0.46	0.64
S85	4.47	4.47	1.24	0.10	−0.04

For the correlation between the FastCAP and T- or C-levels, the asterisks represent significant correlations.

where *x*, behavioral threshold; *y*, FastCAP threshold; *N*, the number of electrodes included in the calculation; *i*, the sequence of electrodes ranging from 1 to *N* to calculate RMSE; and *j*, the sequence of electrodes ranging from 1 to *N* to calculate the mean thresholds. As such, the NRMSE was normalized to the mean standard deviation between the behavioral T-level and FastCAP thresholds. FastCAP thresholds were compared to T- and C-levels using Pearson correlations.

Electrically evoked compound action potential in fast mode thresholds were significantly correlated with T-levels in only 7 out of 20 participants; *r* values were >0.50 in 11/20 participants. FastCAP thresholds were significantly correlated with C-levels in only 6 out of 20 participants; *r* values were >0.50 in 12/20 participants. The different comparisons elicited somewhat different findings. The RMSE, NRMSE, and correlation coefficients were compared using Pearson correlations (Table 4). Significant correlations were observed between RMSE (with DC adjustment) and NRMSE (*r* = 0.71, *p* < 0.001) and the correlation coefficients between FastCAPs and T-levels (*r* = −0.55, *p* = 0.012), between NRMSE and correlation coefficients between FastCAPs and T-levels (*r* = −0.88, *p* < 0.001) or C-levels (*r* = −0.48, *p* = 0.034).

**TABLE 4:** Correlations among the different approaches comparing across-electrode profiles between FastCAP thresholds, behavioral T-levels, and behavioral C-levels.

		RMSE between FastCAP and T-level (with DC adjustment)	NRMSE between FastCAP and T-level	Correlation coefficient FastCAP vs. T-level	Correlation coefficient FastCAP vs. C-level
RMSE between FastCAP and T-level (without DC adjustment)	*r*	0.18	0.00	0.09	−0.10
	*p*	0.458	0.994	0.705	0.668
RMSE between FastCAP and T-level (with DC adjustment)	*r*		0.71	−0.55	−0.31
	*p*		<0.001*	0.012*	0.190
NRMSE between FastCAP and T-level	*r*			−0.88	−0.48
	*p*			<0.001*	0.034*
Correlation coefficient FastCAP vs. T-level	*r*				0.32
	*p*				0.164

The asterisks represent significant correlations.

## 4 Discussion

### 4.1 FastCAP vs. ECAP

The present study suggests that the FastCAP may be an efficient and effective technique for NRM. N1 amplitudes and latencies were significantly correlated between FastCAPs and ECAPs within and across stimulation levels. FastCAP demonstrated good-to-excellent test-retest reliability in terms of N1 latency and amplitude.

We found that the RMSE between the ECAP and FastCAP N1 amplitudes steadily increased with stimulation level. The RMSE between ECAP and FastCAP N1 latencies steadily decreased with stimulation level (Table 1). The steady increase in RMSE between ECAP and FastCAP N1 amplitudes with higher stimulation levels could reflect variability in the neural recruitment patterns and saturation effects. As stimulation levels increase, the neural response may become more nonlinear, leading to greater discrepancies between the methods (Brown et al., 1990; Westen et al., 2011). The steady decrease in RMSE for N1 latencies with increasing stimulation levels may be attributed to differences in stimulating rates between the two methods and less latency variability in higher stimulation (Clay and Brown, 2007).

### 4.2 FastCAP is less time-consuming than ECAP

The time requirement to measure FastCAPs was much less than needed to measure ECAPs. However, this was primarily due to the much faster stimulation rate used in the FastCAP (33.3 Hz) compared to the ECAP (2.5 Hz, the maximum rate in the Nurotron NRM system). Averaging *N* step A, B, C, and D responses is a time-saving measure used by other CI manufacturers.

The data transmission time between the computer and implant is a significant limitation of the overall measurement time. It involves initialization, handshake, data transfer, and verification between the implant, speech processor, and computer. This process is quite time-consuming, with each transmission taking approximately 80 ms in the Nurotron system. In FastCAP, the data for steps A–C are stored in the device hardware and transmitted to the computer after *N* measurements are completed. For *N* iterations, the number of data transmissions is 4 per electrode and level for the FastCAP and 4 × *N* per electrode per level for the ECAP. The execution duration also reduced by measuring step D (noise) one time and then re-using this value. In the forward masking subtraction method, step D represents the measurement without any stimulation, serving as the baseline or noise floor of the measurement system. It is reasonable to assume that the results of step D would remain constant with different stimulating amplitudes. Therefore, in FastCAP, step D is only run once at the first amplitude, and its results are reused for subsequent stimulation amplitudes. Re-using step D values further reduces the execution duration required for each measurement by 25%, compared to the traditional ECAP method.

Other approaches have been introduced to reduce the time required for ECAP measurement. For instance, Garcia et al. (2023) developed “SpeedCAP,” which optimizes the measurement of a full matrix of ECAPs by reusing the A and D steps for each electrode while shifting the B and C steps across the array. This approach reduced the panoramic ECAP measurement time from 45 min (444 ECAP waveforms) to just 8 min (253 ECAP waveforms), with each waveform taking only 1.89 s to record. In comparison, FastCAP required approximately 46.9 s to record 20 ECAP waveforms, averaging 2.34 s per waveform. While SpeedCAP achieves faster data acquisition by recycling measurement steps and focusing on a single current level (C-level), its accuracy can be limited when multiple levels are required for threshold determination in clinical contexts. FastCAP, on the other hand, balances speed with precision by leveraging on-device averaging and robust data transmission protocols, ensuring reliable ECAP thresholds across multiple levels without sacrificing accuracy. This makes it more adaptable to diverse clinical scenarios, such as determining T-levels for CI programming. In terms of reliability, SpeedCAP introduces up to an 8.2% error margin in ECAP amplitudes due to its optimized protocol, which may compromise its applicability in cases requiring high precision. In contrast, FastCAP has demonstrated consistent test-retest reliability, reinforcing its potential for routine clinical use.

### 4.3 FastCAP thresholds vs. behavioral T- and C-levels

Electrically evoked compound action potential thresholds are used by many clinicians to guide programming of T- or C-levels on individual electrodes in speech processor MAPs. This application is particularly useful with infants, young children, and difficult-to-test patients from whom it is difficult or impossible to obtain behavioral responses. Previous studies show that ECAP threshold profiles can give us a clue as to the shape of the T- and C-levels across the array (Zarowski et al., 2020; Franck and Norton, 2021; Holstad et al., 2009; Allam and Eldegwi, 2019), and ECAP thresholds tend to fall above T-levels due to temporal integration and the stimulation rate differences between ECAP (slow single pulses) and behavioral (fast pulse trains) stimuli (McKay et al., 2013; McKay and Smale, 2017; Biesheuvel et al., 2018).

The relationship between ECAP and behavioral thresholds remains unclear (Biesheuvel et al., 2018; de Vos et al., 2018; Sismono et al., 2022). Allam and Eldegwi (2019) discovered a strong positive correlation between C-level and neural response telemetry level measurements (*r* = 0.76), as well as between T-level and neural response telemetry level measurements (*r* = 0.79). Chao et al. (2023) found that ECAP thresholds were strongly correlated with T-levels in children with cochlear nerve deficiency at basal (*r* = 0.55, *p* = 0.002), middle (*r* = 0.70, *p* < 0.001), and apical electrodes (*r* = 0.70, *p* < 0.001). Thangaraj et al. (2023) found a significant correlation between ECAP thresholds to C-levels in 22 pediatric MED-EL CI users (*r* = 0.61, *p* < 0.01).

However, a meta-analysis of previous ECAP studies (de Vos et al., 2018) suggested a weak pooled relationship between ECAP thresholds and T- or C-levels (*r* = 0.58 and *r* = 0.61, respectively). In their analysis, there were many inconsistencies across studies, including age at testing (child vs. adult) (Brown et al., 2010), prelingual vs. postlingual deafness (Alvarez et al., 2010), differences in electrode array type and position in the cochlea (Rajati et al., 2014), the time course of ECAP measurements (intraoperative and postoperative) (Gordon et al., 2004; Brown et al., 2010; Spivak et al., 2011), the time course of T- and C-level measurements (Gordon et al., 2004), electrode stimulation site (e.g., apical, middle, and basal electrodes) (Schvartz-Leyzac and Pfingst, 2018; Sismono et al., 2022; Chakravorti et al., 2019), and ECAP threshold method (e.g., van de Heyning et al., 2016). As such, it is difficult to arrive at a consensus regarding the relationship between ECAP thresholds and T- or C-levels.

Our analysis showed that only NRMSE between FastCAP thresholds and T-levels was predictive of the correlations between FastCAP thresholds and behavioral T- and C-levels (Table 4). Indeed, there was no significant relationship between correlation coefficients between FastCAP thresholds/T-levels FastCAP thresholds/C-levels. This suggests that NRMSE may be a robust predictor of profile similarity between NRMs and behavioral T- or C-levels.

Unfortunately, significant correlations between FastCAP and T-levels were observed in only 35% of participants; significant association between FastCAP and C-levels were observed in only 55% of participants; several issues might contribute to the limited predictive value of FastCAPs for T- and C-levels. First, only five electrodes were tested using only 10 sweeps, which may not be sufficient for correlational analyses. Second, the present results showed no correlation between CI experience and *r* values from the FastCAP vs. T-level correlations (*r* = −0.21, *p* = 0.395). Our findings are not fully aligned with those of Hughes et al. (2001), who observed longitudinal changes in ECAPs and behavioral measures as CI users gained more experience with their devices. While their study did not directly examine the relationship between ECAPs and behavioral measures (T or C levels), a thorough reanalysis of their data reveals that, within the first year following implantation, the correlation between ECAPs and behavioral measures—specifically with C-levels—undergoes significant changes. In contrast, between 1 and 2 years post-implantation, the relationship between ECAPs and both T and C levels appears to stabilize. The present participants had at most 6 months of CI experience, with 45% having CI experience ≤2.4 months. This is much shorter CI experience than in Hughes et al. (2001). It is unclear if the predictive value of FastCAPs for T-levels may change with longer duration of CI experience, where the reliability of T-level measurements may also improve over time. Third, while T- and C-levels may be influenced by age at testing, we did not observe a significant relationship between r values from the FastCAP vs. T-level correlations and age at testing (*r* = 0.25, *p* = 0.279). However, age at test may have been an issue for pediatric participants (S68, S76, S83, S84, and S85), in whom no significant correlations were observed between FastCAP and T- or C-levels.

However, the lack of correlation between FastCAP thresholds and T-levels in most of the present pediatric participants highlights the potential advantages of using NRMs to map T- and C-levels, as the poor correlations may have been partly driven by difficulty in accurately measuring T- or C-levels. It is possible that T- or C-levels derived from NRMs such as the FastCAP may better represent the actual threshold profile across electrodes. Given that FastCAP may overestimate behavioral T-levels, the FastCAP profile could be used to estimate T-levels by stimulating all electrodes in ensemble at a very low level (e.g., near device minimum). The ensemble stimulation would be increased until the patient indicates they heard something. Next, electrodes would be swept for loudness slightly above T-level and adjustments made as necessary. The ensemble would then be adjusted again until obtaining T-level. The advantage of the ensemble stimulation is that it would incorporate the expected multi-channel loudness summation (McKay et al., 2001). After obtaining T-levels, the ensemble stimulation with the adjusted profile would be increased until obtaining C-levels. Again, electrodes would be swept for loudness and adjustments would be made as necessary. The map would then be tested with live speech mode to ensure appropriate loudness. This method is similar to that suggested by Smoorenburg et al. (2002). While this method does rely on patient input, it may be more reliable and efficient than sequentially measuring dynamic ranges on individual electrodes. For patients who cannot indicate threshold even with the suggested ensemble stimulation (e.g., infants and toddlers), the FastCAP profile may be useful to estimate C-level and then T-levels could be set to be a criterion value below C-level. In all these scenarios, the rapidly obtained FastCAP profile could expedite clinical fitting of CIs.

### 4.4 Limitations to the present study

In the present study, FastCAPs were measured for only 5 electrodes, where the mean measurement time across participants was 46.9 s; for all 24 electrodes, FastCAP measurement time would be expected to be 225.1 s. While it is not expected that FastCAP accuracy or measurement time would be affected by the number of electrodes measurements, this should be confirmed. The mean measurement time for ECAPs across participants and the 5 test electrodes was 340.3 s. As discussed previously, transmitting the data from each step (steps A–D) for each iteration of *N* is much more time consuming than transmitting the data from the average of *N* iterations for A, B, C, and D steps. Note also that only 10 sweeps were used for ECAPs and FastCAPs in the present study

Also, the stimulation rate was much higher for FastCAP (33.3 Hz) than for ECAP (2.5 Hz), which requires the lower stimulation rate to allow for the data transmission of each iteration of the A, B, C, and D steps. This largely contributed to the time advantage to the FastCAP. The stimulation rate used for ECAPs has been shown to affect responses, with lower amplitudes associated with higher rates due neural adaptation (Clay and Brown, 2007). The RMSE was used to quantify the average difference in N1 latency and amplitude between different methods or different runs. The mean RMSE of N1 latency between FastCAP and ECAP was 34.2 μs, which was comparable to that between two consecutive runs by FastCAP (29.7 μs). The mean RMSE of N1 amplitude between FastCAP and ECAP was 60.9 μV (or 3.1 dB), which was also comparable to that between two consecutive runs by FastCAP (59.9 μV or 3.1 dB). The data suggest that despite the differences in stimulation rates, the N1 latency and amplitude measured by FastCAP were comparable to those measured by ECAP and exhibited good-to-excellent test-retest reliability, which further demonstrate the reliability of FastCAP.

An IPI of 350 μs between the masker and probe was used to measure FastCAPs and ECAPs. Previous studies have shown that as IPI increases, the ECAP induced by the probe pulse gradually recovers from neural adaptation caused by the masking pulse, resulting in a gradual decrease in amplitude (He et al., 2017). Morsnowski et al. (2006) suggested that values between 300 and 375 μs were appropriate IPIs. However, the optimal IPI may depend on the age at testing. Mussoi and Brown (2019) found that younger CI users (18–40 years) had faster rates of neural recovery than did older CI users (68–82 years). Optimal IPIs for FastCAP should be investigated in the future.

## 5 Conclusion

The FastCAP technique offers a promising solution to reduce time consumption associated with traditional ECAP measurements to users of the Nurotron CI device. FastCAPs were significantly correlated with traditional ECAPs, and exhibited good-to-excellent test-retest reliability. Moreover, FastCAP thresholds were significantly correlated with T- and C-levels. The FastCAP offers an efficient clinical tool to optimize CI processor settings, particularly T-levels, while maintaining accuracy and reliability.

## Data Availability

The datasets presented in this study can be found in online repositories. The names of the repository/repositories and accession number(s) can be found below: Mendeley Data, doi: 10.17632/cxhy5rd3zy.1.
