# Supplementary material for: Preliminary evaluation of the FastCAP for users of the Nurotron cochlear implant
Source: Front Neurosci. 2025 Jan 7;18:1523212. doi: 10.3389/fnins.2024.1523212 (PMC11748202; doi:10.3389/fnins.2024.1523212)
Supplement: Supplementary file 1 [file Supplementary_file_1.docx]

Appendix 1. Technical Details of the Stimulation and Measurement System

**Stimulation Controller**

The stimulation controller regulates the circuit and the switch bank for the stimulating electrodes. The data and commands determine the settings of the following parameters in the stimulation circuit: stimulus amplitude, pulse phase duration, and inter-phase gap. The switch bank also controls additional parameters such as: inter-pulse interval, stimulated electrode, and stimulation rate. These parameters can be adjusted using the NRM test module embedded in the NuroSound fitting software.

**Measurement System**

The measurement system comprises the following components:

Recording Switch Bank: Manages the recording electrodes. Operates independently of the stimulation switch bank, enabling unrestricted selection of both stimulation and recording electrodes. For instance, in Fig. 2, the stimulus electrode is El 4, and the recording electrode is El 5.

Amplifier: Includes a bandpass filter to suppress undesired noise. Offers four selectable gains: 32, 44, 52, and 64 dB.

Auto-Zero Offset Cancellation Circuit: When the cancellation circuit is active, the amplifier output is set to 0. When the circuit is deactivated, the measurement system operates normally.

Analog-to-Digital Converter (ADC): Uses a 12-bit successive approximation register. Provides a maximum sample rate of 250 kHz for converting amplified signals to digital data.

Accumulative Register: Supports efficient data handling for the measurement process.

**System Independence and Flexibility**

The independent operation of the stimulation and recording switch banks ensures flexibility in selecting electrodes without restrictions. This design supports robust and noise-suppressed measurements across various testing scenarios.
