# Supplementary material for: Preliminary evaluation of the FastCAP for users of the Nurotron cochlear implant
Source: Front Neurosci. 2025 Jan 7;18:1523212. doi: 10.3389/fnins.2024.1523212 (PMC11748202; doi:10.3389/fnins.2024.1523212)
Supplement: Supplementary file 2 [file Table_1.docx]

Supplementary Table S1. Participant demographic information for Experiment 1.

| **Participant** | **Sex** | **Age at test**  **(yrs)** | **Dur deaf (yrs)** | **Etiology** | **CI exp**  **(yrs)** | **CI ear** |
| --- | --- | --- | --- | --- | --- | --- |
| S46 | M | 22 | 1 | Sudden | 1.0 | R |
| S47 | F | 38 | 7 | Unknown | 0.4 | R |
| S48 | F | 7 | 2 | Congenital | 0.4 | L |
| S49 | M | 17 | 1 | Unknown | 1.0 | L |
| S50 | F | 29 | 3 | Unknown | 0.5 | L |
| S51 | M | 28 | 2 | Unknown | 0.2 | R |
| S52 | M | 6 | 4 | Congenital | 0.7 | R |
| S53 | F | 30 | 5 | Unknown | 0.6 | L |
| S54 | M | 36 | 2 | Unknown | 0.4 | R |
| S55 | F | 48 | 21 | Unknown | 0.3 | R |
| S56 | M | 1 | 1 | Congenital | 0.1 | R |
| S57 | M | 17 | 2 | Sudden | 0.1 | L |
| S58 | M | 45 | 5 | Unknown | 0.4 | R |
| S59 | F | 42 | 1 | Unknown | 0.5 | L |
| S60 | F | 19 | 2 | Unknown | 0.8 | L |
| S61 | M | 37 | 6 | Sudden | 0.1 | R |
| S62 | M | 4 | 3 | Congenital | 0.1 | L |
| S63 | M | 56 | 14 | Unknown | 0.4 | L |
| S64 | M | 3 | 3 | Congenital | 0.1 | L |
| S65 | M | 4 | 4 | Congenital | 0.5 | L |

F = female; M = male; Dur deaf = duration of deafness; CI exp = CI experience; L = left; R = right.
