# Supplementary material for: Preliminary evaluation of the FastCAP for users of the Nurotron cochlear implant
Source: Front Neurosci. 2025 Jan 7;18:1523212. doi: 10.3389/fnins.2024.1523212 (PMC11748202; doi:10.3389/fnins.2024.1523212)
Supplement: Supplementary file 3 [file Table_2.docx]

Supplementary Table S2. Participant demographic information for Experiment 2.

| **Participant** | **Gender** | **Age at test (yrs)** | **Dur deaf (yrs)** | **Etiology** | **CI exp (yrs)** | **CI**  **ear** |
| --- | --- | --- | --- | --- | --- | --- |
| S66 | F | 42 | 13 | Progressive | 0.3 | L |
| S67 | F | 19 | 5 | Unknown | 0.5 | L |
| S68 | M | 5 | 4 | Congenital | 0.2 | L |
| S69 | M | 56 | 17 | Progressive | 0.3 | L |
| S70 | F | 62 | 24 | Progressive | 0.3 | L |
| S71 | F | 17 | 6 | Unknown | 0.2 | R |
| S72 | F | 16 | 3 | Unknown | 0.2 | L |
| S73 | M | 43 | 13 | Unknown | 0.2 | L |
| S74 | M | 43 | 7 | Unknown | 0.2 | R |
| S75 | F | 42 | 10 | Progressive | 0.2 | R |
| S76 | M | 16 | 8 | Congenital | 0.2 | R |
| S77 | F | 21 | 3 | Sudden | 0.3 | L |
| S78 | M | 21 | 5 | Sudden | 0.6 | R |
| S79 | F | 50 | 8 | Unknown | 0.4 | L |
| S80 | F | 50 | 1 | Unknown | 0.4 | R |
| S81 | M | 26 | 5 | Sudden | 0.5 | L |
| S82 | M | 26 | 4 | Sudden | 0.5 | R |
| S83 | M | 12 | 11 | Congenital | 0.5 | L |
| S84 | M | 8 | 8 | Unknown | 0.2 | L |
| S85 | F | 10 | 2.5 | Congenital | 0.1 | R |

F = female; M = male; Dur deaf = duration of deafness; CI exp = CI experience; L = left; R = right.
